# RANKL-convergent osteoimmune network framework in osteoporosis: integrating macrophage activation, T-cell imbalance, and cytokine crosstalk

**DOI:** 10.3389/fimmu.2026.1911132

**Published:** 2026-07-29

**Authors:** Kaiyuan Zheng, Yufeng Ouyang, Wei Jiang, Siyu Wang, Bing Lan, Nik Nasihah Nik Ramli, Chong Yin

**Affiliations:** 1 Department of Rehabilitation Medicine, Intensive Care Medicine, Department of Clinical Laboratory, Affiliated Hospital of North Sichuan Medical College, Nanchong, Sichuan, China; 2 School of Graduate Studies, Post Graduate Centre, Neuroscience & Mental Well-being Centre (NeuroMIND), International Medical School, Management & Science University, Shah Alam, Selangor, Malaysia; 3 Department of Rehabilitation Medicine, Ziyang Hospital of Traditional Chinese Medicine, Ziyang, Sichuan, China; 4 Department of Rehabilitation Medicine, Affiliated Hospital of Southwest Medical University, Luzhou, Sichuan, China

**Keywords:** cytokine networks, macrophage polarization, osteoimmunology, osteoporosis, RANKL, T-cell subsets

## Abstract

**Background:**

Osteoporosis is classically defined by reduced bone mass and microarchitectural deterioration, but osteoimmune dysregulation within the bone marrow microenvironment is increasingly recognized as an important contributor to bone loss. The RANKL/RANK/OPG axis represents a dominant pathway governing osteoclast differentiation and bone resorption, yet its activity is shaped by macrophage activation, T-cell subset imbalance, and cytokine feedback loops. Existing reviews often examine these components separately; fewer conceptualize their network-level convergence on RANKL-dependent osteoclastogenesis across osteoporosis subtypes and disease stages.

**Methods:**

Literature was searched in PubMed, Web of Science, and Scopus using combinations of “osteoporosis”, “osteoimmunology”, “RANKL”, “macrophage polarization”, “Th17”, “Treg”, “cytokine”, and related terms. Priority was given to mechanistic studies, animal models, clinical observational studies, and recent reviews directly relevant to RANKL-dependent osteoclastogenesis and bone marrow immune regulation.

**Results:**

Pro-inflammatory M1-like macrophages, Th1/Th17 responses, and cytokines such as TNF-α, IL-1β, IL-6, and IL-17 may promote osteoclastogenesis by upregulating RANKL expression, increasing the RANKL/OPG ratio, and enhancing the responsiveness of osteoclast precursors to RANKL. Together, these mechanisms form a pro-inflammatory and pro-osteoclastogenic amplification loop. Conversely, M2-like macrophages, osteal macrophages, Treg/Th2 responses, and factors such as IL-10, TGF-β, IL-4, IL-13, and OPG may restrain excessive osteoclast formation and contribute to inflammation resolution, immune tolerance, and bone repair. Different forms of osteoporosis may share RANKL-dependent bone resorption as a common final effector process, yet differ substantially in their upstream immune drivers.

**Conclusion:**

Interpreting RANKL as a convergent effector node, rather than a RANKL-exclusive explanation, provides a framework for understanding osteoporosis heterogeneity and guiding osteoimmune-based stratification. Future studies incorporating human bone marrow samples, longitudinal cohorts, single-cell and spatial omics, immunometabolic analyses, and bone-targeted delivery technologies are needed to validate network states and translate immunomodulatory strategies into individualized osteoporosis management.

## 1 Introduction

Osteoporosis is a systemic skeletal disorder characterized by reduced bone mass, deterioration of bone microarchitecture, and increased bone fragility. Its major clinical consequences include fragility fractures, chronic pain, impaired mobility, and an increased risk of mortality (1). Traditionally, the central pathological basis of osteoporosis has been considered to involve an imbalance in bone remodeling, in which osteoclast-mediated bone resorption exceeds osteoblast-mediated bone formation, thereby shifting bone homeostasis toward net bone loss (2). However, with the development of osteoimmunology, increasing evidence indicates that the skeletal and immune systems are not independent entities. Instead, they share multiple cell types, cytokines, receptors, and signaling pathways. Dysregulation of immune homeostasis can profoundly contribute to the onset and progression of osteoporosis by modulating osteoclastogenesis, osteoblast function, and the bone marrow microenvironment (3, 4).

Within the osteoimmune regulatory network, the RANKL/RANK/OPG axis, which consists of receptor activator of nuclear factor-κB ligand, its functional receptor RANK, and the soluble decoy receptor osteoprotegerin, not only coordinates osteoclastogenesis but also links immune activation to bone metabolism (5). Upon binding to RANK on the surface of osteoclast precursor cells, RANKL initiates downstream pro-osteoclastogenic signaling pathways, thereby promoting osteoclast differentiation, fusion, maturation, and bone-resorptive activity. In contrast, OPG inhibits RANKL-mediated osteoclastogenesis by competitively binding to RANKL (6, 7). Notably, RANKL is not exclusively derived from cells of the osteoblast lineage and osteocytes. Under inflammatory or immune-activated conditions, immune cells such as activated T cells may also serve as important sources of RANKL (8, 9). Meanwhile, pro-inflammatory cytokines, including TNF-α, IL-1β, IL-6, and IL-17, can further amplify osteoclastogenesis and bone resorption by upregulating RANKL expression, altering the RANKL/OPG ratio, or enhancing the responsiveness of osteoclast precursors to RANKL/RANK signaling (10, 11). Thus, in this context, inflammatory and immune-derived signals converge on the RANKL/RANK/OPG axis and are translated through this pathway into enhanced osteoclast differentiation and bone-resorptive activity. Accordingly, the RANKL/RANK/OPG axis should be viewed not only as a direct effector pathway governing osteoclastogenesis but also a critical convergence point at which immune cells, inflammatory mediators, and bone remodeling processes interact within the bone marrow microenvironment.

Among immune cells implicated in osteoporosis-related osteoimmunology, macrophages and T cells are two of the most extensively studied cell populations and are closely associated with RANKL-dependent osteoclastogenesis (12). Their effects can be summarized at three interrelated levels. First, macrophages exhibit marked plasticity. Their pro-inflammatory or pro-reparative phenotypes can shape either a pro-osteoclastogenic or reparative bone marrow microenvironment by regulating inflammatory cytokine release, the RANKL/OPG balance, and the responsiveness of osteoclast precursors to RANKL signaling (13, 14). Second, imbalances among T-cell subsets can directly or indirectly affect the RANKL axis. Activated T cells may act as inducible sources of RANKL; the Th17/IL-17 axis generally exhibits strong pro-osteoclastogenic potential; whereas Treg-associated responses can restrict excessive osteoclastogenesis through immunosuppressive mediators and cell contact-dependent mechanisms (15, 16). Third, cytokines derived from macrophages, T cells, and other bone marrow cells do not regulate bone resorption in a linear or unidirectional manner. Rather, their effects are determined by cell source, temporal context, local concentration, and accompanying signals, which collectively dictate whether the RANKL axis is amplified, restrained, or regulated in a context-dependent manner (12, 16, 17).

However, previous reviews have often explained the immune mechanisms underlying osteoporosis from a single perspective, such as RANKL signaling, macrophage polarization, T-cell subsets, or cytokine-mediated regulation. In addition, broad RANKL-centered reviews have comprehensively summarized the RANKL/RANK/OPG system across bone metabolism, immune regulation, extra-skeletal tissues, and cancer (18). Although such approaches are useful for clarifying local mechanisms, they may overlook the reciprocal amplification and counter-regulatory interactions among different cellular modules in osteoporosis-specific contexts. Therefore, this review conceptualizes RANKL as a convergent effector node linking macrophage activation states, T-cell subset imbalance, cytokine feedback loops, and osteoporosis subtype-specific bone marrow niche remodeling. By emphasizing network-level interactions rather than isolated mechanisms, and by considering differences among osteoporosis subtypes and disease progression states, we aim to provide an integrated perspective on the pathogenesis of osteoporosis, identify potential regulatory nodes, and discuss the implications of this framework for future immune-related patient stratification and therapeutic development.

## 2 Bone–immune cellular networks in osteoporosis

### 2.1 Overview of the bone marrow immune microenvironment

The bone marrow microenvironment is a central site in the initiation and progression of osteoporosis. It consists of skeletal cells, stromal cells, hematopoietic cells, immune cells, and vascular-associated cells, including osteocytes, osteoblast-lineage cells, osteoclast precursors, mature osteoclasts, bone marrow mesenchymal stem/stromal cells, macrophages, T cells, B cells, endothelial cells, and pericytes. Rather than acting independently, these cells form an interactive regulatory network through direct cell–cell contact, ligand–receptor interactions, cytokines, chemokines, metabolites, and extracellular vesicles (19).

Within this niche, osteocytes sense mechanical, endocrine, and biochemical cues and regulate bone remodeling through molecules such as RANKL, OPG, and sclerostin (20–25). Osteoblast-lineage cells mediate bone matrix formation and also control osteoclast precursor recruitment and differentiation through M-CSF, RANKL, OPG, and chemokines (12). Osteoclast precursors and mature osteoclasts constitute the main bone-resorptive module, while monocyte/macrophage-lineage cells provide osteoclast precursors and regulate inflammation, apoptotic cell clearance, and tissue repair (21, 22). Bone marrow mesenchymal stem/stromal cells influence osteogenesis, adipogenesis, and immune regulation, thereby affecting bone formation and marrow adiposity (23, 24). Endothelial cells and pericytes support the bone marrow niche by maintaining vascular supply, angiogenic signaling, hematopoietic support, and cellular trafficking (24). Immune cells, such as T cells and B cells, further modulate osteoclastogenesis and bone remodeling through cytokine release and RANKL/OPG-related pathways (25).

### 2.2 Pathological remodeling of the bone marrow microenvironment in osteoporosis

Under physiological conditions, the bone marrow microenvironment maintains a dynamic balance among bone resorption, bone formation, and immune regulation. Osteoclasts remove old bone, osteoblast-lineage cells generate new bone matrix, and osteocytes coordinate remodeling by sensing mechanical and metabolic cues (26). Local immune cells also contribute to remodeling homeostasis. Macrophages, particularly bone surface osteomacs, support the osteoblast niche, matrix mineralization, apoptotic cell clearance, inflammation resolution, and bone repair (27–29). Treg cells, Th2-related responses, and immunoregulatory mediators such as IL-10 and TGF-β further restrain excessive inflammation and help maintain the coupling between bone resorption and bone formation (30, 31).

In osteoporosis, estrogen deficiency, aging, oxidative stress, metabolic abnormalities, chronic inflammation, and pharmacological exposure can disrupt this homeostasis (2, 3, 32). The bone marrow niche then shifts from a state supporting coupled remodeling and repair toward one characterized by enhanced osteoclastogenesis, impaired osteogenesis, and insufficient immune regulation (33, 34). This transition is commonly associated with an increased RANKL/OPG ratio, greater abundance or responsiveness of osteoclast precursors, enhanced macrophage- and T-cell-associated inflammatory activity, suppressed osteogenic differentiation, and increased marrow adiposity (35–37). Thus, pathological remodeling in osteoporosis reflects not merely an increase in inflammation or a change in a single cell population, but a broader disruption of cellular composition, differentiation status, spatial organization, and intercellular communication (**Table 1**).

**Table 1:** Major cellular components of the bone marrow osteoimmune microenvironment and their roles in osteoporotic remodeling.

Cell population	Physiological role in the bone marrow niche	Osteoporosis-associated alteration	RANKL/OPG-related role	Representative mediators/references
Osteocytes	Act as mechanosensitive and endocrine-responsive coordinators of bone resorption–formation coupling.	Estrogen deficiency, aging, glucocorticoid exposure, oxidative stress, and osteocyte senescence may shift osteocyte signaling toward a pro-resorptive state.	Form a foundational regulatory layer of RANKL/OPG. Local OPG production restrains RANKL activity, whereas stressed or senescent osteocytes can contribute to RANKL dominance.	RANKL, OPG, sclerostin (19, 37, 38, 93, 95, 98);
Osteoblast-lineage and bone-surface cells	Produce bone matrix, support mineralization, and provide signals required for osteoclast precursor survival and differentiation.	Osteogenic differentiation and mineralization are impaired; bone-surface RANKL-expressing cells adjacent to osteoclasts can promote local activation.	Provide M-CSF, RANKL, OPG, and chemokines. Their spatial proximity to RANK+ precursors helps determine whether RANKL is functionally osteoclastogenic.	M-CSF, RANKL, OPG, chemokines (12, 38);
Bone marrow stromal cells/osteoprogenitors	Maintain osteogenic potential, immune regulation, and the balance between osteogenic and adipogenic lineage commitment.	Reduced osteogenic potential and increased marrow adiposity contribute to impaired bone formation and uncoupled remodeling.	Can modulate the local RANKL/OPG balance and provide stromal support for osteoclastogenesis, especially when inflammatory or adipogenic signals dominate.	RANKL/OPG, adipogenic signals, cytokines (22, 30, 34, 100);
Osteoclast precursors and mature osteoclasts	Represent the monocyte–macrophage lineage effector module responsible for bone resorption.	Precursor number, recruitment, or responsiveness may increase in estrogen deficiency, aging, and inflammatory states.	Express RANK and respond directly to RANKL. RANKL activates TRAF6–NF-κB, MAPK/AP-1, and NFATc1 pathways to drive differentiation, fusion, survival, and resorption.	RANK, TRAF6, NF-κB, MAPK/AP-1, NFATc1 (12, 33, 35, 36);
Macrophages and osteal macrophages	Support efferocytosis, inflammation resolution, bone-surface maintenance, osteoblast support, and tissue repair.	M1-like dominance amplifies inflammation and osteoclastogenesis; insufficient M2/osteomac-associated repair may maintain a pro-resorptive niche.	Regulate RANKL/OPG indirectly by releasing cytokines and by altering the sensitivity of osteoclast precursors to RANKL.	TNF-α, IL-1β, IL-6, IL-10, TGF-β, extracellular vesicles (20, 21, 26, 27, 43, 44);
T cells	Provide adaptive immune surveillance and immunoregulatory control of bone remodeling.	Activated T cells and Th17/Treg imbalance are common in postmenopausal and inflammatory bone loss; Th1 responses are context dependent.	Activated T cells and Th17 cells may supply RANKL and IL-17, whereas Treg/Th2 responses restrain RANKL responsiveness and support repair.	RANKL, IL-17, TNF-α, IFN-γ, IL-10, TGF-β, IL-4, IL-13, CTLA-4 (15, 39, 40, 51, 52, 73, 76);
B cells	Contribute to immune surveillance and bone mass maintenance, partly through OPG production under homeostatic conditions.	In aging, estrogen deficiency, or chronic inflammation, disease-associated B-cell subsets may shift toward a RANKL-producing phenotype.	Context-dependent regulator: OPG source in homeostasis but inducible RANKL source in disease.	OPG, RANKL, CXCR4 (41, 42);
Endothelial cells, pericytes, and vascular niche cells	Provide oxygen, nutrients, angiogenic cues, hematopoietic support, and channels for cell trafficking.	Vascular niche injury and reduced reparative capacity are implicated in long-standing and age-related osteoporosis.	Do not primarily function as direct RANKL effectors, but shape the spatial organization of immune-cell trafficking, osteogenesis, and remodeling-unit repair.	Angiogenic signals, vascular niche cues, trafficking signals (23, 93, 100, 101);
Accessory innate immune cells	Participate in innate surveillance, antigen presentation, and inflammatory relay within the bone marrow niche.	Specific inflammatory or aging-associated subsets may contribute to niche degeneration or adaptive immune activation.	Mostly regulate the RANKL-centered network indirectly by shaping cytokine release, antigen presentation, and Th1/Th17-associated inflammation.	IL-12, IL-23, TGF-β1+CCR5+ neutrophil signals (18, 31, 47);

## 3 RANKL-convergent osteoimmune network

### 3.1 Multicellular sources of RANKL/OPG

**Figure 1:**
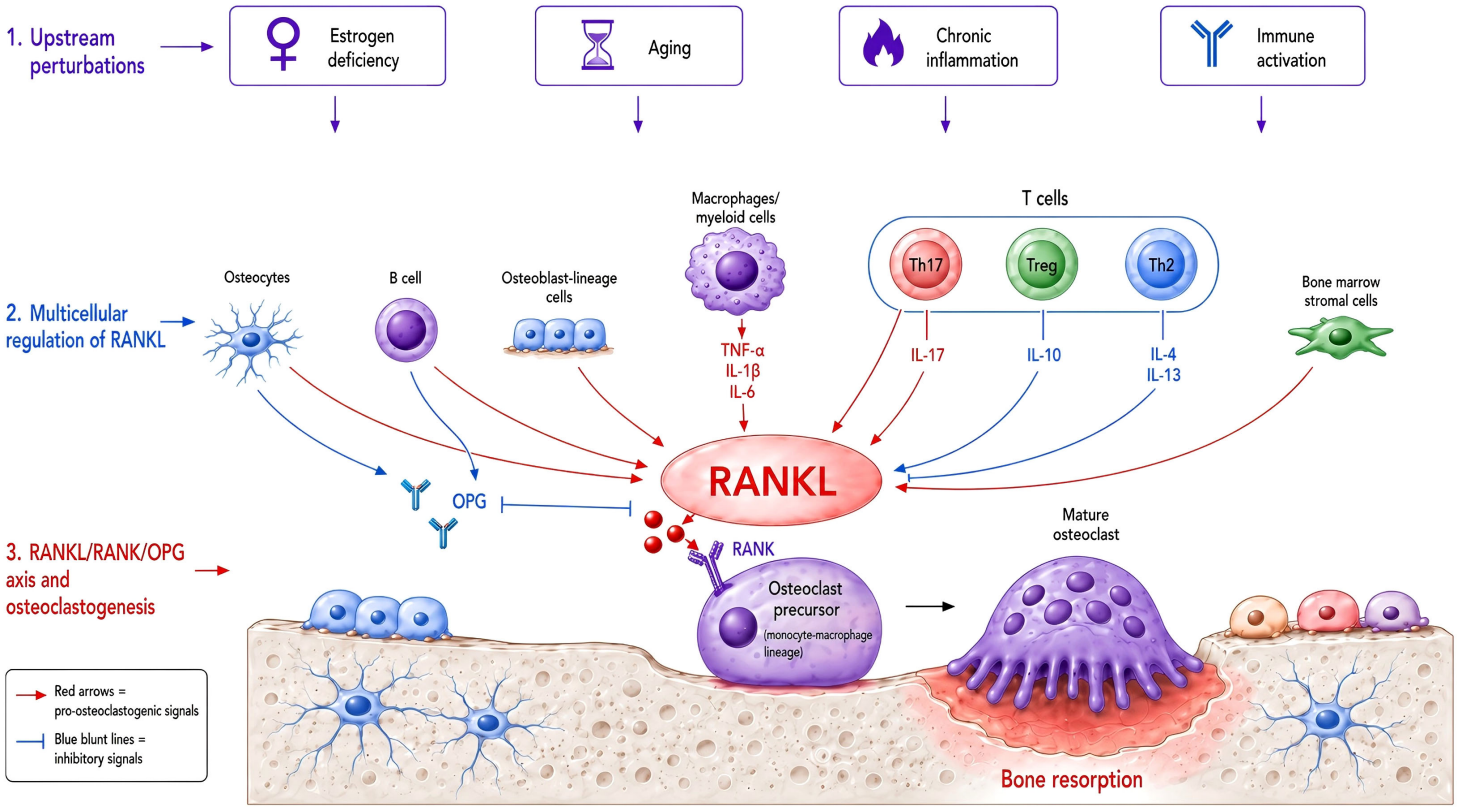
RANKL-convergent osteoimmune network regulating osteoclastogenesis. Bone marrow stromal cells, osteoblast-lineage cells, macrophage-lineage cells, B-cells and T-cell subsets converge on RANKL regulation under estrogen deficiency, aging, chronic inflammation, and immune activation. RANKL activates RANK on osteoclast precursors to drive bone resorption, whereas OPG and anti-inflammatory cytokines restrain this process; red arrows indicate activation and blue blunt lines indicate inhibition.

RANKL can be produced by multiple cell types, including osteocytes, osteoblast-lineage cells, bone marrow stromal cells, and immune cells such as activated T cells and B cells (38–41), whereas RANK is predominantly expressed on osteoclast precursors of the monocyte-macrophage lineage (18). Upon binding to RANK, RANKL activates downstream signaling pathways, including TRAF6-mediated NF-κB, MAPK/AP-1, and NFATc1 pathways, thereby driving osteoclast precursor differentiation, cell fusion, cytoskeletal remodeling, maturation of bone-resorptive function, and survival maintenance (12). OPG acts as a soluble decoy receptor for RANKL and competitively blocks RANKL–RANK binding, thereby limiting osteoclastogenesis (**Figure 1**). Recent succinylome profiling has further suggested that RANKL may participate in metabolic reprogramming of osteoclasts by modulating the activity of mitochondrial metabolic enzymes, thus supporting the energy demands required for osteoclast differentiation and bone resorption (42).

RANKL/OPG control is spatially organized and context dependent. During steady-state bone remodeling, osteocytes, osteoblast-lineage cells, bone-lining cells, reversal-phase cells, canopy/lumen cells, and bone marrow stromal populations provide important local skeletal-niche sources of RANKL and OPG (41). Recent single-cell transcriptomic analyses combined with *in situ* hybridization have localized Tnfsf11/RANKL expression to osteoprogenitor populations at or near remodeling bone surfaces, including bone-lining cells, reversal cells, and canopy/lumen cells (43). Independent immunolocalization studies have likewise detected RANKL protein in bone-lining cells (44) and in reversal and canopy cells at osteoclast-containing surfaces (45). This feature indicates that the pro-osteoclastogenic effect of RANKL is determined not only by its overall expression level but also by its spatial relationship with osteoclast precursors, mature osteoclasts, and OPG-producing cells.

However, under conditions of estrogen deficiency, aging, chronic inflammation, or autoimmune activation, the relative contribution of immune-cell-derived and inflammation-associated signals may increase. Among them, Th17 cells may directly provide RANKL and, through IL-17, induce stromal cells such as osteoblasts and synovial fibroblasts to upregulate RANKL expression, thereby forming a key bridge between T-cell activation and bone destruction (46). Conversely, Treg cells, Th2-associated responses, and immunoregulatory mediators such as IL-10 and TGF-β can limit excessive osteoclastogenesis by suppressing pro-inflammatory cytokine release, reducing RANKL expression, or enhancing OPG-mediated inhibitory effects (47). The role of B cells is context dependent. Under homeostatic conditions, B cells contribute to bone mass maintenance by producing OPG; however, in the context of estrogen deficiency, chronic inflammation, or aging, specific B-cell subsets may shift toward a pro-osteoclastogenic phenotype, produce RANKL, and promote osteoclast formation (48, 49). In addition, macrophages and other myeloid cells can influence RANKL/OPG expression and the sensitivity of osteoclast precursors to RANKL signaling by regulating pro-inflammatory mediators such as TNF-α, IL-1β, and IL-6 (13).

Taken together, osteoporosis reflects a context-dependent reweighting of a multicellular RANKL/OPG network. In physiological remodeling, RANKL and OPG are locally coordinated by skeletal, stromal, and immune components to maintain coupled bone resorption and formation. In osteoporotic or inflammatory conditions, estrogen deficiency, aging, chronic inflammation, and immune activation may increase local RANKL availability, weaken OPG-mediated restraint, and enhance osteoclast precursor responsiveness. Therefore, the RANKL/OPG axis is best understood as a major convergence hub for osteoclastogenic signaling within the osteoimmune microenvironment, rather than as the sole regulator of osteoporotic remodeling.

### 3.2 M1-like/Th1/Th17 pro-inflammatory crosstalk and RANKL-dependent osteoclastogenesis

Under conditions of estrogen deficiency, aging, oxidative stress, metabolic dysregulation, or chronic inflammatory stimulation, myeloid cells in the bone marrow may shift toward an M1-like state. M1-like macrophages are characterized by increased expression of molecules such as iNOS, TNF-α, IL-1β, IL-6, IL-12, and IL-23 (50, 51). These cytokines can directly act on osteoclast precursors of the monocyte–macrophage lineage and enhance their sensitivity to RANKL signaling. They can also act on osteocytes, osteoblast-lineage cells, bone marrow stromal cells, and inflammation-associated mesenchymal cells to promote RANKL expression or weaken OPG-mediated restraint (52, 53).

Meanwhile, M1-like macrophages can shape T-cell responses through cytokine-mediated mechanisms. IL-12 secreted by M1-like macrophages promotes Th1 differentiation, whereas IL-1β, IL-6, and IL-23 participate in Th17 differentiation, stabilization, and IL-17 release (54, 55). In osteoimmune regulation, Th17 cells link adaptive immunity to bone resorption. First, Th17 cells themselves can express membrane-bound or soluble RANKL (56). Second, IL-17 secreted by Th17 cells can induce RANKL upregulation in multiple stromal cell types (46). Third, IL-17 can induce the release of TNF-α, IL-1β, and IL-6, forming an “IL-17–pro-inflammatory cytokine–RANKL” amplification loop (57). The role of Th1 cells is context dependent. Th1 cells can produce TNF-α and IFN-γ. TNF-α promotes the survival, proliferation, and differentiation of osteoclast precursors and cooperates with RANKL to amplify pro-osteoclastogenic signaling pathways (53). IFN-γ exerts dual effects. IFN-γ can promote TRAF6 degradation and thereby inhibit RANKL-induced osteoclast differentiation. However, under immune-activated conditions, IFN-γ can enhance antigen presentation and T-cell activation, indirectly increasing T-cell-derived RANKL and TNF-α and ultimately exerting a pro-resorptive effect (58, 59).

Conversely, Th1 and Th17 cells can reciprocally maintain and amplify the inflammatory phenotype of M1-like macrophages. IFN-γ produced by Th1 cells is a classical signal for M1-like activation through STAT1-associated transcriptional programs. TNF-α can synergize with IFN-γ to activate inflammatory pathways further sustaining the M1-like state of macrophages (60, 61). IL-17A derived from Th17 cells can also directly act on macrophages, inducing the production of TNF-α, IL-1β, and IL-6 and enhancing MAPK-, NF-κB-, and AP-1-related signaling, thereby promoting the maintenance of or shift toward an M1-like inflammatory phenotype (62, 63). Therefore, Th1 and Th17 cells are not merely downstream consequences of M1-like macrophage induction but also important reciprocal drivers that sustain M1-like inflammatory activation.

On the basis of this bidirectional crosstalk, the RANKL–RANK axis is further amplified (35, 64). In the ovariectomized models, Th17/Treg imbalance is associated with increased osteoclast activity, while estrogen deficiency can elevate the M1-/M2-like ratio (50, 65). In summary, the key significance of M1-like/Th1/Th17 crosstalk explains how innate and adaptive immunity cooperate through cytokine-driven positive feedback to continuously convert chronic low-grade inflammation or local immune activation into RANKL-dependent osteoclastogenesis.

### 3.3 The M2-like/Treg/Th2 regulatory module and osteoimmune repair

Corresponding to the M1-like/Th1/Th17 pro-inflammatory amplification module, Treg cells and Th2-associated cytokines can promote macrophage polarization toward an M2-like anti-inflammatory and pro-reparative phenotype, thereby forming a regulatory module associated with immune tolerance, inflammation resolution, and tissue repair (66, 67). First, M2-like macrophages secrete IL-10, TGF-β, and chemokines such as CCL17 and CCL22. These mediators not only suppress local M1-like inflammatory activation, but also promote the recruitment, expansion, and functional maintenance of Treg cells and contribute to the development of Th2-type immune responses (68, 69). Specifically, IL-10 inhibits macrophages and antigen-presenting cells from producing pro-inflammatory mediators, including IL-1β, IL-6, TNF-α, IL-12, and IL-23, thereby weakening the inflammatory conditions required for Th1/Th17 cell differentiation (70). TGF-β participates in the maintenance of immune tolerance and, under specific cytokine conditions, promotes Treg cell stability (71, 72). In addition to soluble cytokines, M2-like macrophage-derived exosomes may also influence T-cell differentiation. In malignant pleural effusion, these exosomes were reported to promote Treg differentiation, potentially through miR-4443; however, this mechanism has not yet been directly validated in osteoporosis or osteoimmune models (73).

Second, Treg cells and Th2 responses can reciprocally maintain and reinforce the anti-inflammatory and reparative phenotype of M2-like macrophages. IL-10 and TGF-β secreted by Treg cells can suppress NF-κB-related inflammatory gene expression in macrophages, reduce the levels of pro-inflammatory mediators such as TNF-α, IL-1β, and IL-6, and promote the shift of macrophages from an M1-like inflammatory state toward an M2-like regulatory state (74–76). Meanwhile, Treg cells can also reduce the co-stimulatory capacity of antigen-presenting cells through contact-dependent mechanisms such as CTLA-4, thereby further limiting sustained Th1/Th17 cell activation (76, 77). IL-4 and IL-13 derived from Th2 cells are key factors in maintaining the reparative M2-like macrophage state. Through the IL-4Rα–STAT6 signaling pathway, these cytokines promote the expression of M2-associated molecules such as Arg1, CD206, Ym1, and Fizz1, thereby enhancing the anti-inflammatory activity, apoptotic cell clearance, and tissue-repair functions of macrophages (78, 79).

On the basis of this bidirectional interaction, RANKL–RANK-mediated osteoclastogenic signaling is inhibited at multiple levels. IL-10 secreted by M2-like macrophages and Treg cells can suppress key transcriptional programs in osteoclast precursors, including NF-κB, c-Fos, and NFATc1, thereby reducing their responsiveness to RANKL. Treg cells can also limit osteoclast precursor maturation through TGF-β- and CTLA-4-dependent mechanisms (80, 81). IL-4 and IL-13 secreted by Th2 cells not only promote M2 macrophage polarization, but also inhibit the expression of RANKL and pro-inflammatory mediators (82, 83). In addition, the M2-like/Treg/Th2 module helps suppress Th1/Th17 activation and reduce IL-17A- and TNF-α-mediated inflammatory T-cell activation, thereby attenuating osteoclast-associated responses (69, 84). In ovariectomized mice and models of inflammatory bone loss, strategies that promote M2-like macrophage polarization or enhance Treg/Th2-associated anti-inflammatory immune responses can alleviate the Th17/Treg imbalance, suppress local inflammatory mediators, improve the RANKL/OPG balance, and ultimately reduce osteoclast activity (65, 85).

It should be emphasized that the M2-like/Treg/Th2 module should not be simply interpreted as a uniformly beneficial cellular combination. During the early phase of bone repair, a moderate inflammatory response contributes to the clearance of damage-associated signals and the initiation of repair. A timely subsequent transition toward M2-like/Treg/Th2-dominated inflammation resolution and tissue reconstruction is more conducive to the restoration of osteoimmune homeostasis (86). Conversely, if pro-inflammatory responses persist while M2-like macrophage-, Treg-, and Th2-associated regulatory signals remain insufficient, the bone marrow microenvironment may remain in a chronic pro-inflammatory and pro-osteoclastogenic state. The role of TGF-β also highlights this context dependence: in an immune-tolerant environment, TGF-β supports Treg cell stability and tissue repair; however, in the presence of pro-inflammatory mediators such as IL-6, IL-1β, and IL-23, it may also participate in Th17 differentiation (87, 88).

**Figure 2:**
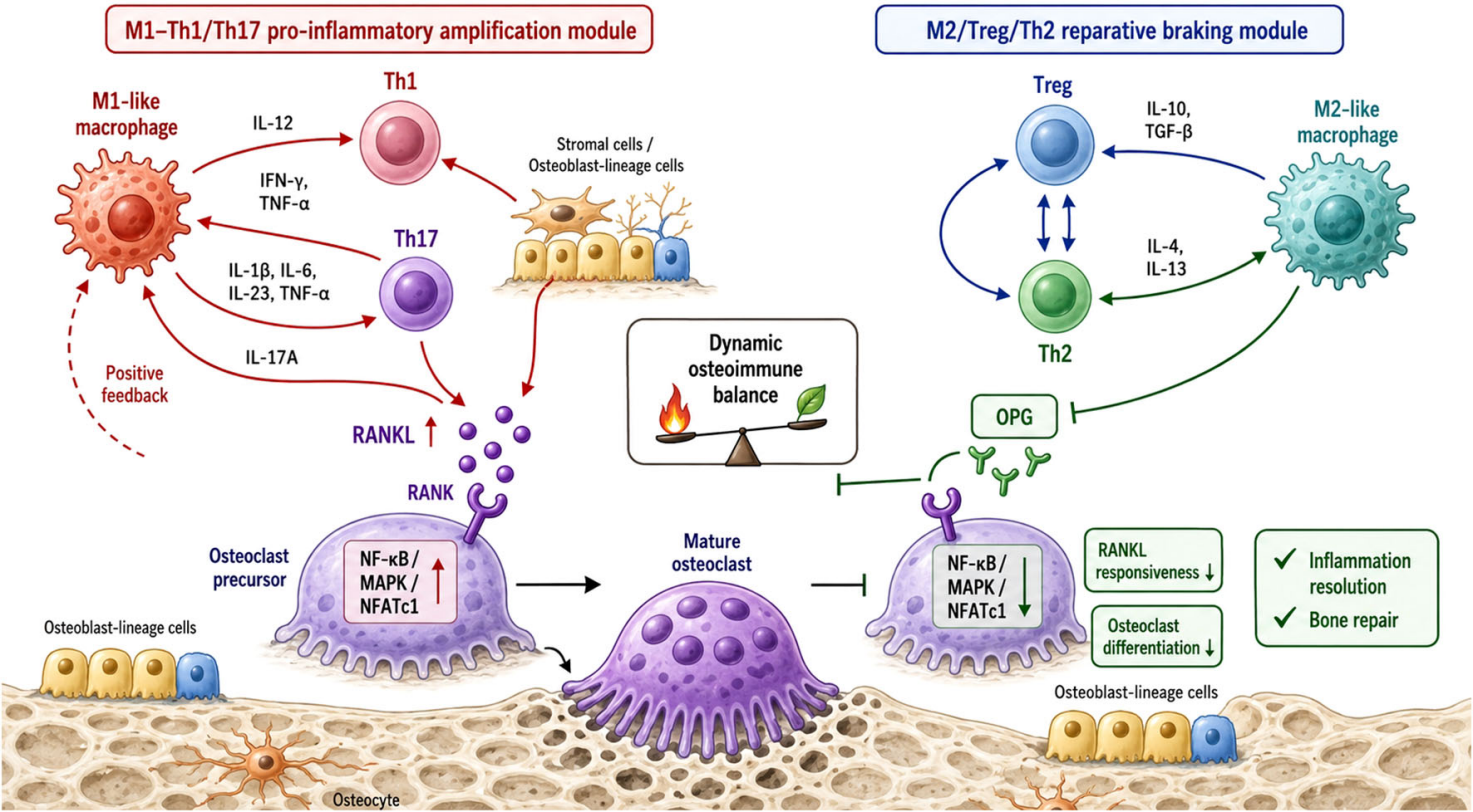
Bidirectional regulation of M1-like/Th1/Th17 and M2-like/Treg/Th2 modules in RANKL-dependent osteoclastogenesis. The M1-like/Th1/Th17 module amplifies inflammatory cytokine signaling and enhances RANKL–RANK–NF-κB/MAPK/NFATc1 activation, thereby promoting osteoclastogenesis and bone resorption. In contrast, the M2-like/Treg/Th2 module suppresses RANKL responsiveness through IL-10, TGF-β, IL-4, IL-13, and OPG, shifting the bone marrow microenvironment toward inflammation resolution and bone repair; arrows indicate promotion, whereas blunt-ended lines indicate inhibition.

In summary, the M2-like/Treg/Th2 regulatory module represents an important counterbalancing system within the RANKL-convergent osteoimmune network. Its major functions are to reduce the burden of pro-inflammatory cytokines, limit RANKL/OPG imbalance, attenuate the responsiveness of osteoclast precursors to RANKL, and promote the transition of the bone marrow microenvironment from inflammatory bone resorption toward inflammation resolution and bone repair (**Figure 2**).

### 3.4 Integration of cytokine networks: pro-osteoclastogenic, anti-osteoclastogenic, and context-dependent signals

Accumulating evidence indicates that cytokine networks serve an integrative role in linking macrophage polarization, T-cell subset imbalance, and the RANKL axis. The pro-osteoclastogenic network is typically composed of TNF-α, IL-1β, IL-6, IL-17, IL-23, and chemokines such as CCL2 and CCL3 (89). Within this network, TNF-α and IL-1β are inflammatory amplifiers positioned closest to the RANKL effector arm (90–92). IL-17 plays a bridging role in this network by converting Th17-associated adaptive immune activation into upregulated RANKL expression. At the same time, IL-17 promotes the release of inflammatory mediators such as TNF-α, IL-1β, and IL-6, thereby forming an “IL-17–inflammatory cytokine–RANKL” amplification loop (93). IL-23 primarily sustains and stabilizes Th17 responses and supports IL-17 production, whereas IL-6 promotes osteoclastogenesis both indirectly by programming Th17 differentiation and through sIL-6R-dependent trans-signaling in osteoblast-lineage cells, which activates STAT3 and induces RANKL expression (94, 95). Chemokines such as CCL2 and CCL3 primarily recruit monocytes, osteoclast precursors, and inflammatory cells to bone resorption sites, thereby increasing the local supply of RANKL-responsive target cells and strengthening the spatial effects of RANKL signaling (10). Together, these signals promote an increased RANKL/OPG ratio, inflammatory priming of osteoclast precursors, and enhanced bone resorption.

Corresponding to the pro-inflammatory amplification network, the anti-osteoclastogenic and reparative network is mainly composed of OPG, IL-10, IL-4, IL-13, and TGF-β. OPG is the most direct endogenous inhibitor of the RANKL axis. IL-10 exerts both anti-inflammatory and anti-osteoclastogenic effects (96, 97). As Th2-associated cytokines, IL-4 and IL-13 promote an M2-like reparative immune environment and suppress RANKL-induced osteoclast differentiation through STAT6-related mechanisms. In addition, both cytokines can increase OPG expression and reduce the expression of RANKL and RANK (83, 98, 99).

Notably, the functions of some cytokines are highly context dependent. IFN-γ can directly inhibit RANKL-induced osteoclast differentiation by promoting TRAF6 degradation; however, during sustained immune activation, it may indirectly promote RANKL- and TNF-α-associated bone resorption by enhancing T-cell responses (58, 59). TGF-β supports Treg cells and tissue repair in an immune-tolerant environment, but may promote Th17 differentiation in the presence of IL-6, IL-1β, and IL-23 (71, 100). These observations suggest that cytokine function is not determined by the molecule itself in isolation, but by its position within the network and the accompanying signals in the local microenvironment.

In summary, cytokine networks can be understood as a multilayered regulatory system of the RANKL axis. When pro-inflammatory amplification signals remain dominant, the RANKL/OPG balance shifts toward osteoclastogenesis. When anti-inflammatory and reparative signals are restored, the RANKL axis is restrained, and the bone marrow microenvironment is more likely to transition toward inflammation resolution and bone repair (**Table 2**).

**Table 2:** Macrophage/T-cell/cytokine modules converging on RANKL-dependent osteoclastogenesis.

Network module/layer	Major sources or targets	Core mediators	Effect on the RANKL/RANK/OPG axis	Net skeletal effect	Context notes/references
TNF-α/IL-1β proximal inflammatory amplification	M1-like macrophages, activated T cells, osteoclast precursors, osteocytes, stromal cells	TNF-α, IL-1β	Synergizes with RANKL, increases RANKL expression, lowers the threshold for RANKL-induced osteoclastogenesis, and helps maintain mature osteoclast activity.	Enhanced osteoclast survival, differentiation, and bone resorption.	Most relevant when inflammation persists near bone remodeling units (45, 46, 83, 84, 99, 117, 118);
IL-6/IL-23 Th17-maintenance layer	M1 macrophages, antigen-presenting cells, stromal cells, activated T-cell environments	IL-6, IL-23	Maintains and stabilizes Th17/IL-17 responses that indirectly increase RANKL expression.	Sustained adaptive immune support for RANKL-dependent osteoclastogenesis.	Represents an upstream maintenance layer rather than a direct terminal effector (47, 48, 86, 91);
Th17/IL-17 adaptive-immune bridge	Th17 cells; osteoblast-lineage cells; stromal cells; synovial or periodontal fibroblast-like cells in inflammatory disease	IL-17A, T-cell-derived RANKL	Th17 cells can express RANKL directly; IL-17 induces stromal RANKL and inflammatory cytokines, forming an IL-17–cytokine–RANKL loop.	Strong pro-osteoclastogenic conversion of T-cell activation into bone resorption.	Prominent in postmenopausal and inflammatory bone loss (39, 49, 50, 85, 92, 119);
M1–Th1/Th17 positive-feedback loop	M1 macrophages, Th1 cells, Th17 cells, osteoclast precursors	IL-12, IL-1β, IL-6, IL-23, TNF-α, IFN-γ, IL-17	M1 cytokines promote Th1/Th17 differentiation; Th1/Th17 cytokines reinforce M1 activation and expand RANKL-related inflammation.	Self-amplifying pro-inflammatory and pro-osteoclastogenic network.	Explains how chronic low-grade inflammation is converted into RANKL-dependent osteoclastogenesis (32, 43, 47, 53–56, 58);
Chemokine spatial organization layer	Macrophages, T cells, stromal cells, inflammatory bone marrow niche	CCL2, CCL3, CXCL10	Recruit monocytes, osteoclast precursors, and inflammatory cells to sites where RANKL is locally available.	Increases the local pool of RANKL-responsive target cells and amplifies spatial bone resorption.	Important for local niche organization rather than only cytokine concentration (10, 32, 57);
OPG decoy restraint layer	Osteocytes, osteoblast-lineage cells, osteogenically active osteoblasts, B cells	OPG	Competitively binds RANKL and prevents RANKL–RANK interaction.	Direct endogenous inhibition of osteoclastogenesis and bone resorption.	Loss of OPG restraint shifts the RANKL/OPG ratio toward osteoclastogenesis (5–7, 38, 41);
IL-10 anti-inflammatory and anti-osteoclastogenic layer	M2-like macrophages, Treg cells, tolerogenic antigen-presenting cells	IL-10	Suppresses TNF-α, IL-1β, IL-6, IL-12, and IL-23 production; directly inhibits NF-κB, c-Fos, and NFATc1 programs in osteoclast precursors.	Reduces precursor responsiveness to RANKL and attenuates osteoclast maturation.	Links inflammation resolution with inhibition of RANKL effector signaling (63, 87, 88);
Treg cell regulatory arm	Treg cells, antigen-presenting cells, osteoclast precursors, MSCs	IL-10, TGF-β, CTLA-4	Restrains co-stimulation and pro-inflammatory T-cell activation; suppresses osteoclast precursor maturation and RANKL responsiveness.	Limits excessive osteoclastogenesis and may support osteogenic differentiation.	Treg activity is protective when regulatory function is preserved (69, 70, 73, 74, 111);
Th2–M2 reparative reinforcement	Th2 cells, M2-like macrophages, osteoblast-lineage cells	IL-4, IL-13	Promotes M2-like polarization and STAT6-dependent inhibition of RANKL-induced osteoclastogenesis; can increase OPG and reduce RANKL/RANK expression.	Suppresses inflammatory bone resorption and supports repair-oriented immunity.	Protective when coordinated with appropriate timing of inflammation resolution (71, 72, 76, 89, 90);
IFN-γ context-dependent switch	Th1 cells, activated T cells, macrophage-rich inflammatory niches	IFN-γ	Directly inhibits RANKL-induced osteoclast differentiation by promoting TRAF6 degradation, but may indirectly increase T-cell activation, RANKL, and TNF-α.	Anti- or pro-resorptive depending on whether direct osteoclast inhibition or immune activation dominates.	Should not be classified as uniformly anti-osteoclastogenic (51, 52);
TGF-β context-dependent switch	Treg cells, macrophages, bone cells, inflammatory microenvironment	TGF-β	Supports Treg stability and tissue repair in tolerogenic contexts, but can contribute to Th17 differentiation when IL-6, IL-1β, and IL-23 are present.	May promote repair or contribute to osteoclastogenic inflammation depending on accompanying cytokines.	Highlights why cytokine effects must be interpreted at the network level (64, 80, 81, 91);

### 3.5 Osteoimmune network states across disease stages and osteoporosis subtypes

**Figure 3:**
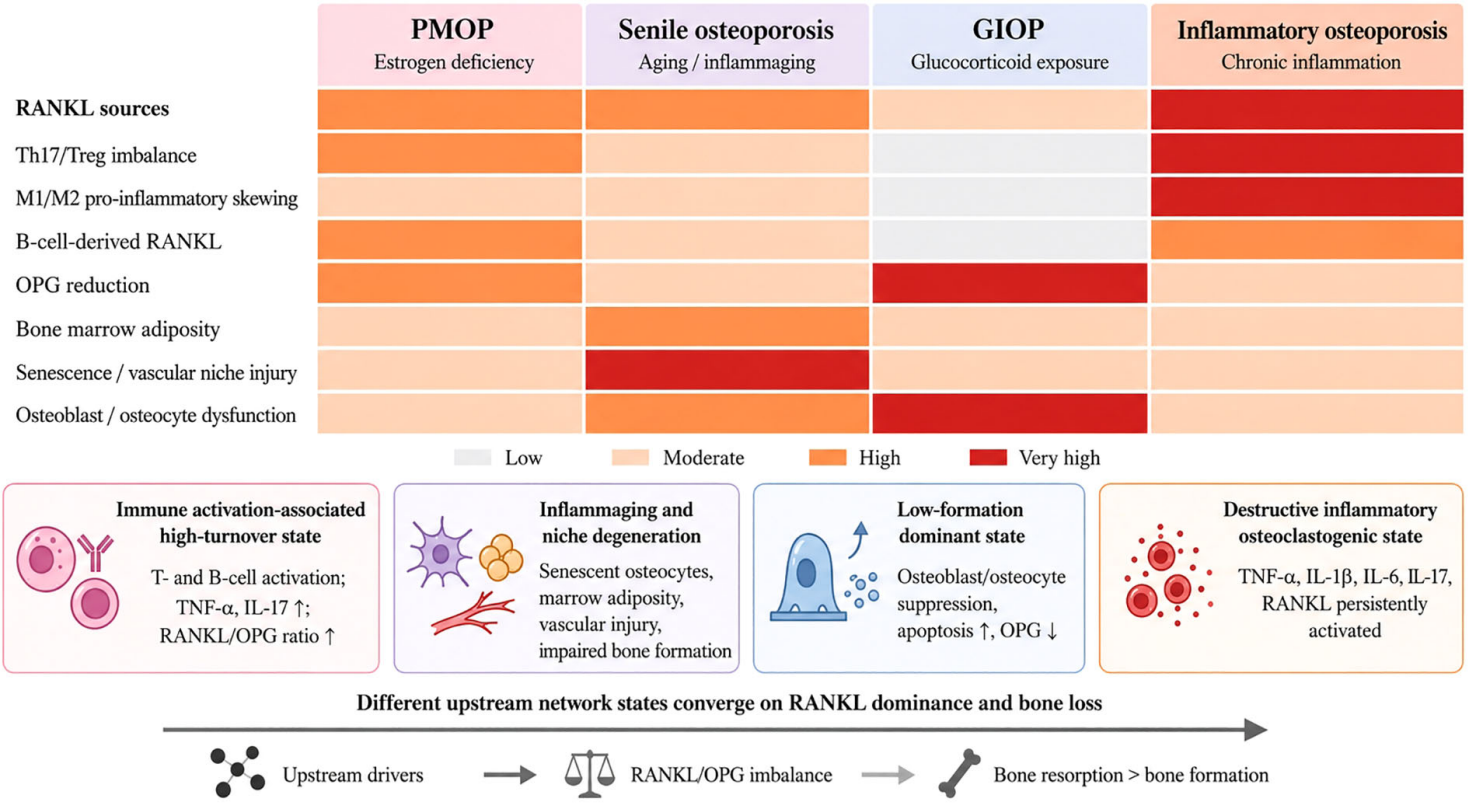
Osteoimmune network states across osteoporosis subtypes. The heatmap compares the relative intensity of key osteoimmune features in PMOP, senile osteoporosis, GIOP, and inflammatory osteoporosis. Distinct upstream drivers—estrogen deficiency, aging/inflammaging, glucocorticoid exposure, and chronic inflammation—shape different immune–niche states, but ultimately converge on RANKL/OPG imbalance and bone resorption exceeding bone formation.

A RANKL-convergent network framework does not imply that all forms of osteoporosis are driven by identical immune mechanisms or exclusively mediated through RANKL. Different subtypes may share RANKL-dependent osteoclastogenesis as a common terminal pathway, but their upstream drivers, immune cell composition, and cytokine profiles are not necessarily the same. Studies suggest that postmenopausal osteoporosis is primarily driven by estrogen deficiency and is characterized by T- and B-cell activation, Th17/Treg imbalance, elevated TNF-α and IL-17 levels, and an increased RANKL/OPG ratio. It therefore represents a typical immune activation-associated high-turnover state (35, 101). Age-related osteoporosis is closely associated with inflammaging, immunosenescence, cellular senescence and the associated senescence-associated secretory phenotype (SASP), and progressive deterioration of the bone marrow microenvironment (25). One hallmark of this deterioration is age-related bone marrow adiposity, characterized by bone marrow adipose tissue (BMAT) expansion and closely associated with reduced bone mass (102, 103). Together, osteocyte senescence, impaired osteoprogenitor function, BMAT expansion, and age-related deterioration of the marrow vascular niche disrupt bone remodeling and contribute to age-related bone loss (25, 37, 104). Glucocorticoid-induced osteoporosis does not necessarily present as a typical pro-inflammatory state. Its core pathology is more often reflected by suppression of osteoblast and osteocyte function, increased apoptosis, decreased OPG expression, and alteration of the RANKL/OPG balance (105, 106). Inflammation-associated osteoporosis or inflammatory bone destruction is characterized by persistent activation of TNF-α, IL-1β, IL-6, IL-17, and RANKL. In this setting, the M1-like/Th1/Th17 pro-inflammatory module is markedly dominant, and both osteoclast precursor recruitment and osteoclast differentiation are substantially amplified (107, 108) (**Figure 3**).

From the perspective of disease progression, osteoimmune abnormalities in different forms of osteoporosis usually do not manifest as overt bone mass loss from the outset. Instead, they undergo a continuous process from the accumulation of upstream stimuli, to the establishment of RANKL dominance, and finally to uncoupled bone remodeling. In the early stage of disease, estrogen deficiency, age-associated inflammation, glucocorticoid exposure, or chronic inflammatory stimulation may first trigger immune cell activation, osteocyte stress, and changes in the bone marrow microenvironment, placing osteoclast precursors in a sensitized state with increased responsiveness to RANKL (109). As the disease progresses, the RANKL/OPG balance gradually shifts toward RANKL dominance. Inflammatory mediators such as TNF-α, IL-1β, IL-6, and IL-17 can further amplify osteoclastogenesis and bone resorption by inducing RANKL expression, enhancing osteoclast precursor recruitment, or lowering the threshold for osteoclast differentiation (11, 110). In long-standing disease, abnormal bone remodeling is no longer limited to enhanced osteoclast activity. It is often accompanied by reduced osteoblast differentiation and mineralization capacity, impaired osteocyte regulatory networks, bone marrow adiposity, and vascular niche dysfunction, ultimately resulting in an uncoupled state characterized by increased bone resorption and insufficient bone formation (111, 112).

Therefore, the osteoimmune network state in osteoporosis should be evaluated by integrating the cellular sources of RANKL, the intensity of the M1-like/Th1/Th17 pro-inflammatory module, the functional status of the M2-like/Treg/Th2 reparative module, the degree of cellular senescence and bone marrow niche degeneration, and the disease phase. Such network-based stratification may help explain interpatient differences in responses to anti-RANKL, anti-inflammatory, osteoanabolic, or sequential therapies, and may provide a more mechanistically grounded classification framework for precision intervention in osteoporosis (**Table 3**).

**Table 3:** Osteoimmune network states across osteoporosis subtypes and disease stages.

Osteoporosis subtype or disease stage	Dominant upstream driver	Major osteoimmune/niche alteration	RANKL/OPG and remodeling phenotype	Therapeutic implication	Representative references
Postmenopausal osteoporosis	Estrogen deficiency and sex hormone-associated immune activation.	T- and B-cell activation, Th17/Treg imbalance, increased TNF-α and IL-17, and altered gut–bone immune trafficking.	Increased RANKL/OPG ratio and high-turnover, immune activation-associated bone resorption.	Anti-RANKL therapy is mechanistically aligned; exploratory strategies may target Th17/Treg balance, IL-17, TNF-α, gut microbiota, or cytokine networks.	(32, 58, 92, 102, 117, 119, 121–123)
Age-related osteoporosis	Inflammaging, immunosenescence, cellular senescence, and degeneration of the bone marrow niche.	Senescent osteocytes and degenerative skeletal stem/stromal niches; marrow adiposity, vascular niche injury, and disease-associated immune-cell subsets.	RANKL dominance may coexist with insufficient osteogenesis, adipogenic drift, and uncoupled remodeling.	May require combined approaches targeting resorption, osteoanabolism, senescence-associated signals, adiposity, and niche restoration.	(18, 24, 31, 33, 42, 93, 100, 101)
Glucocorticoid-induced osteoporosis	Chronic glucocorticoid exposure and direct skeletal-cell toxicity.	Suppressed osteoblast and osteocyte function, apoptosis of osteoblasts/osteocytes, reduced OPG, and altered osteocyte-derived RANKL.	Often characterized by uncoupled remodeling with impaired formation; RANKL-mediated resorption remains actionable but is not the only driver.	Anti-RANKL therapy can increase BMD; osteoanabolic or sequential strategies may be needed when formation failure dominates.	(94, 95, 103)
Inflammation-associated osteoporosis/inflammatory bone destruction	Persistent autoimmune, periodontal, infectious, or local inflammatory stimulation.	Dominant M1–Th1/Th17 module; sustained TNF-α, IL-1β, IL-6, IL-17, and RANKL; recruitment of osteoclast precursors.	Marked RANKL-dependent osteoclastogenesis, aggressive local resorption, and bone erosion.	Requires control of upstream inflammatory disease plus consideration of RANKL blockade or cytokine/network-level intervention.	(96, 97, 104, 114)
Early osteoimmune activation phase	Estrogen deficiency, aging-related inflammation, glucocorticoids, metabolic stress, or chronic inflammation.	Immune activation, osteocyte stress, microenvironmental remodeling, and sensitization of osteoclast precursors.	RANKL dominance may be emerging before severe bone mass loss is clinically evident.	Potential window for risk stratification using bone turnover markers, cytokine profiles, immune phenotyping, and imaging.	(98, 124, 126, 127)
Progressive RANKL-dominant phase	Sustained inflammatory or endocrine–metabolic perturbation.	Amplification of TNF-α, IL-1β, IL-6, IL-17, chemokine recruitment, and expansion of RANKL-responsive precursors.	RANKL/OPG ratio shifts toward RANKL; osteoclast differentiation and bone resorption accelerate.	Anti-RANKL or antiresorptive therapy is central; upstream immune modulation may be considered in selected inflammatory phenotypes.	(11, 39, 45, 46, 83, 99, 102)
Long-standing uncoupled phase	Persistence of upstream perturbation with exhaustion or failure of repair programs.	Reduced osteoblast differentiation and mineralization, impaired osteocyte regulatory networks, marrow adiposity, vascular niche dysfunction, and inadequate M2–Treg/Th2 repair.	Bone loss is driven by both excessive resorption and insufficient formation.	Combination, sequential, or phenotype-guided strategies may be more appropriate than single-node inhibition alone.	(79, 100, 101, 120, 128, 129)

## 4 Therapeutic implications and translational opportunities

### 4.1 RANKL-targeted therapy

RANKL blockade currently represents the most clinically established osteoimmunological targeted strategy. Denosumab, a monoclonal antibody against RANKL, neutralizes RANKL and prevents its interaction with RANK, thereby suppressing osteoclast formation, survival, and bone resorption. In the FREEDOM trial, subcutaneous administration of denosumab every six months significantly reduced the risks of vertebral, non-vertebral, and hip fractures in women with postmenopausal osteoporosis (113). Importantly, the therapeutic relevance of RANKL targeting is not restricted to primary osteoporosis. In glucocorticoid-induced osteoporosis, denosumab produced greater increases in bone mineral density at the lumbar spine and hip than risedronate, suggesting that RANKL-mediated osteoclast activation remains an actionable pathway even under immunosuppressive conditions (114). In inflammatory bone destruction, such as rheumatoid arthritis, Fc-OPG reduced osteoclast numbers and prevented bone erosion in collagen-induced arthritis (115), whereas recombinant OPG protected articular bone in adjuvant-induced arthritis (116). Separately, a neutralizing anti-RANKL monoclonal antibody ameliorated inflammation-associated bone loss in collagen-induced arthritis mice (117).

Nevertheless, RANKL-targeted therapy also highlights the limitations of single-target intervention. Clinically, discontinuation of denosumab may be followed by rebound increases in bone turnover and an elevated risk of vertebral fractures, indicating the need for sequential antiresorptive therapy and long-term management strategies (118, 119). This may be because denosumab primarily inhibits osteoclastogenesis and bone resorption, but does not directly correct persistent pro-inflammatory macrophage polarization, Th17/Treg imbalance, or chronic inflammatory cytokine networks. These observations suggest that, although RANKL blockade is the most mature strategy in osteoimmunological therapy, it cannot fully substitute for upstream modulation of the immune microenvironment.

### 4.2 Immune cell modulation: macrophage reprogramming and T-cell balance

Immune-cell modulation should be positioned as a translational opportunity. Macrophage reprogramming is conceptually attractive because macrophage activation influences inflammation, osteoclastogenesis, osteogenesis, angiogenesis, and repair. The therapeutic goal is not permanent M1 suppression or sustained M2 enhancement, but a phase-appropriate transition from early inflammatory clearance to later reparative remodeling (120). Preclinical studies suggest that inducing an M1-to-M2 transition, using M2 macrophage-derived extracellular vesicles, or modifying their miRNA/metabolite cargo can reduce osteoclastogenic signaling and support osteogenic repair (121–123). These approaches should be clearly distinguished from established antiresorptive therapy because most evidence remains experimental.

T-cell modulation is similarly promising but still exploratory. Restoring Th17/Treg balance, limiting IL-17-associated RANKL induction, or enhancing Treg-mediated suppression can improve bone parameters in OVX or inflammatory arthritis models and inhibit osteoclast differentiation *in vitro* (46, 92, 124–126). Beyond direct regulation of T cells, the gut microbiota and short-chain fatty acids can influence bone formation by modulating Treg function, providing a new indirect route for osteoimmunological intervention (127, 128).

However, these macrophage- and T cell-directed therapeutic strategies should still be approached with caution. On the one hand, macrophage and T-cell subsets play essential physiological roles in host defense, immune tolerance, and tissue repair, and long-term or systemic manipulation may carry immunological risks. On the other hand, most current evidence remains at the level of mechanistic studies, animal models, or extrapolation from inflammatory diseases, and therefore cannot yet be directly regarded as a mature therapeutic option for primary osteoporosis.

### 4.3 Cytokine- and network-level interventions

Cytokine- and network-level interventions target upstream inflammatory drivers that feed into the RANKL axis. In estrogen deficiency models, T-cell-derived TNF-α, IL-1 signaling, and Th17/IL-17 activation contribute to osteoclastogenic bone loss, while blockade of IL-1 or IL-17 can attenuate bone resorption and partially restore OPG/RANKL balance (129–131). These findings support cytokine pathways as modulators of osteoimmune network state, but they do not by themselves establish routine cytokine blockade as an osteoporosis treatment.

Broader network interventions include JAK inhibition and gut microbiota/metabolite modulation. JAK inhibitors act downstream of multiple cytokine receptors and have been reported to increase bone mass in OVX and inflammatory arthritis models, partly through effects on osteoblast function as well as immune signaling (132). Gut microbiota-directed interventions, including probiotics and short-chain fatty acid-related pathways, may influence intestinal barrier integrity, Th17/Treg balance, TNF-α, IL-17, IL-10, and RANKL/OPG regulation, thereby linking systemic immune tone with bone marrow remodeling (133–135).

Taken together, network-level strategies expand the therapeutic concept from isolated inhibition of bone resorption to correction of upstream immune-marrow dysregulation. Their clinical value will depend on defining target populations, demonstrating local bone marrow effects, and establishing safe combinations with antiresorptive, anabolic, or sequential regimens.

### 4.4 Patient stratification, safety, and translational barriers

A key prerequisite for osteoimmunological therapy is the identification of the dominant immune abnormalities in individual patients. Age, sex, and bone mineral density alone are insufficient to determine whether a patient is suitable for immunomodulatory intervention (136). Future strategies should integrate bone turnover markers, inflammatory cytokine profiles, peripheral immune phenotypes, local bone marrow samples, imaging features, and clinical risk factors to establish osteoimmune-based patient stratification. For example, patients with an inflammatory phenotype characterized by marked elevations in Th17/IL-17 and TNF-α may be more suitable candidates for exploratory anti-inflammatory or T-cell-modulating strategies. In contrast, elderly patients dominated by impaired osteogenesis and bone marrow adiposity may require approaches focused on osteoanabolism, anti-senescence, and niche restoration.

The major translational barriers include four aspects. First, immune indicators in peripheral blood may not accurately reflect local pathological changes within the bone marrow. Second, most mechanistic evidence is derived from cellular or animal models, which remain distant from the complexity of human osteoporosis. Third, long-term systemic immunomodulation raises safety concerns, particularly in elderly patients and individuals with chronic comorbidities. Fourth, local osteoimmune biomarkers capable of guiding treatment selection are still lacking. Addressing these challenges will require coordinated efforts involving human bone marrow samples, longitudinal clinical cohorts, spatial omics technologies, and well-designed clinical trials.

## 5 Discussion and future directions

### 5.1 Advantages of the RANKL-convergent network framework

The traditional remodeling model remains useful, but it cannot fully explain why estrogen deficiency, aging, chronic inflammation, metabolic stress, and drug exposure produce overlapping yet heterogeneous patterns of bone loss (137). A RANKL-convergent osteoimmune framework addresses this gap by linking upstream immune and niche disturbances to a shared osteoclastogenic effector pathway.

This framework has three main advantages. First, it explains why diverse upstream stimuli can converge on increased osteoclastogenesis through greater RANKL availability, altered OPG restraint, or enhanced precursor sensitivity. Second, it integrates macrophages, T cells, cytokines, stromal cells, osteocytes, and osteoblast-lineage cells into a communication network rather than treating them as independent mechanisms. Third, it explains why RANKL blockade is effective but may not fully correct upstream inflammatory, senescent, or niche-degenerative states.

Importantly, this is a RANKL-convergent, not RANKL-exclusive, model. Some immune and cytokine-driven processes are partially RANKL independent or act mainly on osteoblasts, osteocytes, stromal-cell fate, vascular niches, or marrow adiposity. RANKL should therefore be regarded as a dominant osteoclastogenic convergence node, rather than the sole mediator of osteoimmune regulation in osteoporosis. Taken together, this subtype- and stage-aware network perspective provides an integrated framework for understanding how context-dependent immune and bone-marrow disturbances converge on osteoclastogenesis in osteoporosis.

### 5.2 Disease heterogeneity and network-state stratification

An important advantage of a network-based explanatory framework is its ability to account for differences among disease subtypes and disease stages. Therefore, osteoporosis should not be stratified solely according to bone mineral density, age, sex, or clinical etiology. Future classification systems should incorporate information on network states, including the predominant sources of RANKL, the balance between pro-inflammatory and pro-resolving immune modules, osteoclast precursor sensitivity, bone turnover markers, inflammatory cytokine profiles, bone marrow adiposity, senescence-associated signals, and imaging-based evidence of skeletal deterioration. Such stratification may help identify which patients are biologically more suitable for anti-RANKL therapy, anti-inflammatory immunomodulation, bone-forming treatment, senescent cell clearance, strategies aimed at restoring the bone marrow niche, or sequential and combination therapeutic regimens.

### 5.3 Limitations of binary models: M1-/M2-like and Th17/Treg are not the whole answer

Although M1-/M2-like macrophage polarization and Th17/Treg imbalance are useful for clarifying the directionality of osteoimmune responses, they should not be regarded as complete or fixed explanations for osteoporotic bone loss. Macrophages exist along a continuum of activation states, and their phenotypes are jointly shaped by cytokines, metabolic substrates, oxidative stress, senescence-associated signals, tissue injury, and the temporal phase of repair. In particular, bone-associated macrophages, or osteomacs, cannot simply be classified as classical M2-like cells. Their importance lies in their spatial localization and their supportive role at bone-forming surfaces (138–140).

T-cell biology is similarly context dependent. A higher Th17/Treg ratio is associated with postmenopausal and inflammatory bone loss, but it may not represent the dominant mechanism in all forms of osteoporosis. IFN-γ produced by Th1 cells has dual effects, exerting direct anti-osteoclastogenic activity while also indirectly promoting inflammation. Th2 cells generally suppress osteoclastogenesis and promote M2-like polarization. CD8+ T cells, γδ T cells, and natural killer T cells may also exert opposite effects under different inflammatory conditions (47, 59, 83). Therefore, future studies should move beyond static lineage labels and define immune-cell function according to activation state, spatial localization, ligand–receptor interactions, metabolic programs, and the relationship of these cells with local bone remodeling units.

### 5.4 Differences between the local bone marrow niche and peripheral immune markers

Many current clinical studies infer the immune status of osteoporosis from the Th17/Treg ratio in peripheral blood, M1-/M2-like markers, or serum cytokine levels. These indicators may reflect systemic inflammation or immunosenescence, but they do not necessarily represent local pathological changes within the bone marrow. The key pathological processes of osteoporosis occur in the bone marrow cavity, on trabecular bone surfaces, at the endosteum, and around bone remodeling units, where cellular composition and cytokine concentrations may differ substantially from those in peripheral blood (141, 142).

This discrepancy represents one of the major barriers in translational osteoimmunology. Future studies should therefore make greater use of human bone marrow aspirates, bone tissues obtained during fracture surgery or joint arthroplasty, longitudinal cohorts, and paired peripheral–local immune profiling. These approaches will be essential for determining whether a specific immune signature is a causal driver, a compensatory repair response, or merely a bystander marker of skeletal aging and systemic disease.

### 5.5 Future technological directions: single-cell analysis, spatial omics, immunometabolism, and targeted delivery

Several technological and translational directions are expected to advance this field. Single-cell RNA sequencing, single-cell ATAC sequencing, mass cytometry, spatial transcriptomics, multiplex immunofluorescence, and spatial proteomics can help define the heterogeneity, spatial localization, and communication patterns of bone marrow immune cells and skeletal cells (143, 144). These approaches may help identify which cell populations provide functionally relevant RANKL, which cells produce OPG within local bone remodeling niches, and how the states of macrophages, T cells, stromal cells, osteocytes, and osteoblast-lineage cells change across disease stages and in response to treatment.

Immunometabolism represents another important direction. Osteoclast differentiation requires mitochondrial activity, oxidative phosphorylation, amino acid utilization, and epigenetic remodeling, and these processes may be influenced by inflammatory cytokines, macrophage-derived extracellular vesicles, aging, and metabolic stress (123, 145). Integrating metabolic profiling with cellular communication maps may help clarify why the same cytokine can produce different skeletal effects depending on the local nutrient environment, redox status, and differentiation state of osteoclast precursors or mesenchymal stromal cells.

Finally, targeted delivery technologies provide an important translational platform. Bone surface–affinity peptides, hydroxyapatite-binding materials, engineered exosomes, nanocarriers, and locally sustained-release hydrogels may deliver immunomodulatory factors more precisely to the bone marrow or bone remodeling regions, thereby reducing systemic immune-related adverse effects (146, 147). However, these strategies should be developed together with reliable biomarkers, patient stratification systems, and clinically relevant endpoint measures. The next stage of osteoimmunology research should move beyond describing isolated immune abnormalities and instead test whether specific network states can predict fracture risk, therapeutic response, rebound phenomena after discontinuation of antiresorptive agents, and the likelihood that patients will benefit from combination or sequential therapy. In this sense, the RANKL-convergent network framework provides a conceptual bridge between mechanistic osteoimmunology and precision management of osteoporosis (**Table 4**).

**Table 4:** Osteoimmune-directed therapeutic strategies and translational considerations in osteoporosis.

Strategy	Main network target	Evidence level in the current review	Mechanistic rationale/expected skeletal effect	Potentially suitable network phenotype	Major limitations and future direction
RANKL blockade, including denosumab and experimental OPG-Fc/anti-RANKL antibodies	Terminal RANKL–RANK effector arm of osteoclastogenesis.	Clinically established for postmenopausal osteoporosis; clinical evidence in glucocorticoid-induced osteoporosis; preclinical evidence in inflammatory bone destruction.	Neutralizes RANKL, suppresses osteoclast formation, survival, and resorption, and reduces fracture risk or inflammatory bone erosion depending on context.	Patients with RANKL-dominant high-turnover bone loss, postmenopausal osteoporosis, glucocorticoid-induced osteoporosis, or inflammatory bone destruction with active osteoclastogenesis.	Does not directly correct upstream immune imbalance. Denosumab discontinuation may cause rebound bone turnover and vertebral fracture risk; planned sequential antiresorptive management is required (102–106).
Macrophage reprogramming and M2 macrophage-derived extracellular vesicles	M1–M2 transition, inflammatory resolution, macrophage–osteoblast/osteoclast communication.	Mainly *in vitro* and animal-model evidence.	Timely M1-to-M2 transition may suppress osteoclastogenesis while supporting osteogenic differentiation, angiogenesis, and repair; M2-derived EVs may deliver regulatory miRNAs, mRNAs, or metabolites.	Inflammatory or repair-defective phenotypes with persistent M1-like activation and insufficient M2/osteomac-associated repair.	Macrophages are essential for host defense and early repair; sustained systemic manipulation may be risky. Bone-targeted or phase-specific delivery is needed (77, 78, 107–110).
T-cell balance modulation, including Th17 inhibition and Treg enhancement	Th17/Treg balance, T-cell-derived RANKL, IL-17, Treg immunosuppressive function.	Mechanistic, co-culture, OVX model, and inflammatory arthritis model evidence.	Reduces IL-17- and RANKL-mediated osteoclastogenesis; Treg cells can inhibit osteoclast differentiation and may support MSC osteogenic differentiation.	Postmenopausal or inflammatory phenotypes with high Th17/IL-17 activity, low Treg function, or elevated Th17/Treg ratio.	Systemic T-cell manipulation may affect immune defense and tolerance. Requires careful phenotyping and safety monitoring before clinical translation in primary osteoporosis (39, 111–114).
Cytokine-targeted intervention, including TNF-α, IL-1, and IL-17 pathways	Upstream inflammatory amplifiers that increase RANKL expression or precursor sensitivity.	Strong mechanistic and animal-model evidence; disease-specific clinical extrapolation from inflammatory disorders.	May reduce RANKL induction, inflammatory priming of osteoclast precursors, and local bone resorption.	Inflammation-dominant phenotypes with elevated TNF-α, IL-1β, IL-17, and active M1–Th1/Th17 modules.	Broad immunomodulation may not be suitable for non-inflammatory or formation-defective osteoporosis; target population and combination regimen remain unclear (83–86, 117–119).
JAK inhibition and network-level cytokine pathway modulation	Integrative downstream signaling node shared by multiple cytokine pathways.	Preclinical and translational model evidence in OVX-induced bone loss and inflammatory arthritis.	May increase bone mass by modulating immune signaling and stimulating osteoblast function, not merely by suppressing inflammation.	Patients or models with cytokine-network activation and combined immune–bone remodeling abnormalities.	Potential for broad immune effects; long-term safety, dose, skeletal endpoints, and combination with standard osteoporosis therapy require clarification (120).
Gut microbiota, probiotics, and short-chain fatty acid-based intervention	Gut–bone immune axis; intestinal barrier, Th17/Treg balance, TNF-α, IL-17, RANKL/OPG.	Animal-model and mechanistic evidence; emerging translational relevance.	May reduce intestinal barrier dysfunction, systemic/bone marrow inflammation, Th17 expansion, and RANKL/OPG imbalance; SCFAs may support bone formation through Treg–WNT10B-related mechanisms.	Hormone-deficiency-associated or inflammation-associated phenotypes linked to gut barrier dysfunction and immune dysbiosis.	Likely adjunctive rather than stand-alone therapy. Human strain specificity, dose, duration, biomarkers, and fracture/BMD endpoints require validation (58, 115, 116, 121–123).
Bone-targeted delivery technologies, including engineered extracellular vesicles, nanocarriers, bone-affinity materials, and hydrogels	Local bone marrow or bone-surface delivery of immunomodulatory and osteoreparative signals.	Emerging preclinical and translational-platform evidence.	May concentrate regulatory molecules near remodeling units, reduce systemic immune exposure, and combine anti-inflammatory, antiresorptive, and osteogenic repair actions.	Patients requiring local or niche-directed intervention, especially where systemic immunomodulation is unsafe.	Requires reliable bone-targeting specificity, controllable release, safety testing, companion biomarkers, and clinically meaningful endpoints (131, 132).
Osteoimmune-based patient stratification and companion diagnostics	Identification of dominant RANKL source, cytokine profile, immune-cell state, bone turnover, local marrow niche, and imaging phenotype.	Conceptual and emerging biomarker framework supported by clinical-marker and omics studies.	Does not directly treat bone loss but guides selection of anti-RANKL, anti-inflammatory, osteoanabolic, anti-senescence, microbiota-based, or sequential approaches.	Heterogeneous osteoporosis populations where BMD, age, sex, or clinical etiology alone are insufficient to choose therapy.	Peripheral blood may not reflect local marrow pathology. Requires paired peripheral–local profiling, longitudinal cohorts, spatial omics, and validated treatment-response biomarkers (124, 126–129).

## 6 Conclusion

Osteoporosis reflects not only uncoupled bone resorption and formation but also remodeling of the local osteoimmune communication network within the bone marrow. The RANKL/RANK/OPG axis remains a dominant effector pathway for osteoclastogenesis, but its activity is shaped by macrophage activation, T-cell subset distribution, cytokine feedback, stromal/osteocyte signals, and disease-stage-specific niche changes.

The central implication of this review is that RANKL should be interpreted as a convergent effector node rather than an isolated pathogenic factor. This perspective helps explain osteoporosis heterogeneity, supports subtype- and stage-specific network stratification, and clarifies why therapies targeting RANKL, inflammation, bone formation, senescence, or the marrow niche may need to be combined or sequenced according to patient biology.

Future research should validate osteoimmune network states using human bone marrow samples, longitudinal cohorts, paired peripheral-local immune profiling, single-cell and spatial omics, immunometabolism, and bone-targeted delivery technologies. Such work may move the field from descriptive mechanisms toward individualized prevention and treatment of osteoporosis.
